# Comparative performance of the Mbita trap, CDC light trap and the human landing catch in the sampling of *Anopheles arabiensis*, *An. funestus *and culicine species in a rice irrigation in western Kenya

**DOI:** 10.1186/1475-2875-4-7

**Published:** 2005-01-25

**Authors:** Evan M Mathenge, Gedion O Misiani, David O Oulo, Lucy W Irungu, Paul N Ndegwa, Tom A Smith, Gerry F Killeen, Bart GJ Knols

**Affiliations:** 1 International centre of Insect Physiology and Ecology, Mbita Point Research and Training Centre, Mbita Point, Kenya; 2 Department of Zoology, University of Nairobi, Nairobi, Kenya; 3 Department of Public Health and Epidemiology, Swiss Tropical Institute, Basel Switzerland; 4 Ifakara Health Research and Development Centre, Ifakara, United Republic of Tanzania; 5 Entomology Unit, International Aatomic Energy Agency, Seibersdorf, Austria

## Abstract

**Background:**

Mosquitoes sampling is an important component in malaria control. However, most of the methods used have several shortcomings and hence there is a need to develop and calibrate new methods. The Mbita trap for capturing host-seeking mosquitoes was recently developed and successfully tested in Kenya. However, the Mbita trap is less effective at catching outdoor-biting *Anopheles funestus *and *Anopheles arabiensis *in Madagascar and, thus, there is need to further evaluate this trap in diverse epidemiological settings. This study reports a field evaluation of the Mbita trap in a rice irrigation scheme in Kenya

**Methods:**

The mosquito sampling efficiency of the Mbita trap was compared to that of the CDC light trap and the human landing catch in western Kenya. Data was analysed by Bayesian regression of linear and non-linear models.

**Results:**

The Mbita trap caught about 17%, 60%, and 20% of the number of *An. arabiensis*, *An. funestus*, and culicine species caught in the human landing collections respectively. There was consistency in sampling proportionality between the Mbita trap and the human landing catch for both *An. arabiensis *and the culicine species. For *An. funestus*, the Mbita trap portrayed some density-dependent sampling efficiency that suggested lowered sampling efficiency of human landing catch at low densities. The CDC light trap caught about 60%, 120%, and 552% of the number of *An. arabiensis*, *An. funestus*, and culicine species caught in the human landing collections respectively. There was consistency in the sampling proportionality between the CDC light trap and the human landing catch for both *An. arabiensis and An. funestus*, whereas for the culicines, there was no simple relationship between the two methods.

**Conclusions:**

The Mbita trap is less sensitive than either the human landing catch or the CDC light trap. However, for a given investment of time and money, it is likely to catch more mosquitoes over a longer (and hence more representative) period. This trap can therefore be recommended for use by community members for passive mosquito surveillance. Nonetheless, there is still a need to develop new sampling methods for some epidemiological settings. The human landing catch should be maintained as the standard reference method for use in calibrating new methods for sampling the human biting population of mosquitoes.

## Background

Mosquito sampling is a prerequisite to most vector population studies [1]. The entomological parameter being studied and the behaviour of the mosquito species being sampled determine the choice of a sampling method [2]. However, most of the available mosquito sampling methods may not allow for such rational choices to be made, as there are major limitations associated with their use [3]. Therefore, new tools for sampling mosquito vector populations must be continuously developed. Nonetheless, even these new sampling tools must be calibrated against the existing ones in different vectorial systems [4] if they are to be adopted for conventional use. A new trap, the Mbita trap has been developed [5] and separately evaluated in quite different vectorial systems in Western Kenya and Madagascar [6,7] with varying degrees of success. The Mbita trap was originally developed in semi-field systems with an *Anopheles gambiae *colony originating from southern Tanzania [5] and proved a sensitive and representative way to sample *An. gambiae*, *Anopheles arabiensis *and *Anopheles funestus *in Western Kenya [6,7]. In sharp contrast, the Mbita trap proved highly insensitive for catching *An. funestus and An. arabiensis *in rice-growing communities in the highlands of Madagascar [6,7]. In this study, the performance of the Mbita trap compared to the CDC light trap hung adjacent to a human-occupied bednet and the human landing catches in the sampling of *An. arabiensis An. funestus *and culicines species of mosquitoes in a rice-growing community in western Kenya with relatively high mosquito densities is reported.

## Methods

### Description of the study area

These studies were carried out in a village adjacent to the Ahero rice irrigation scheme in western Kenya. Populations of *An. arabiensis and An. funestus *[8] as well as culicine species [1] are predominant in this area. The characteristics of the mosquito population and malaria vectorial system in this area have been described in detail elsewhere [1,9].

#### Sampling

In Ahero, three houses were selected upon receiving consent from the household heads. Occupants were given a non-impregnated bed net per sleeping space and trained in their correct use. With informed consent, three young men who had earlier been trained in mosquito sampling [6] were recruited to act as bait in the three alternative mosquito collection methods. On each experimental night, one of the three subjects slept in the Mbita trap (BNT), another slept in a bed net with a CDC light trap suspended beside it (CDC) and the third conducted a human landing catch (HLC) [3]. Both the Mbita trap and the CDC light trap-bed net system were set on mattresses placed on mats laid on the floor and not on beds. In all the experiments, a standard miniature CDC light trap (Model 512; John W. Hock Company, Gainesville, Florida, USA) with an incandescent light bulb was used. The trap was hung beside the bed net on the foot side of the sleeping person with its shield touching the side of the net and its inlet about 25 cm above the sleeping person [10]. Each of the three sampling methods was allocated to one of the three houses on a given night in a 3 × 3 randomised Latin square experimental design replicated 3 times. The human baits did not move around the sites so that the effects of a particular site and the attractiveness of the human bait associated with it were combined for simplified statistical analysis. Sampling was carried out from 20.00 hrs to 06.00 hrs between October and November 2002.

### Ethical considerations

Informed consent was obtained from all the participants. Thick and thin blood smears were regularly taken from the participants to examine for the presence of malaria parasites and, when found positive, they were treated with pyrimethamine-sulfadoxine (Fansidar^®^). A follow-up was made to ensure that any parasitaemia was fully cleared. If parasitaemia did not clear, the participants were referred to hospital for further treatment with second line drugs. The Kenya Medical Research Institute (KEMRI) through the KEMRI/National Ethical Review Committee granted ethical approval (KEMRI/7/3/1) for this study.

### Mosquito processing

Mosquitoes were taken to the laboratory and killed by suffocation with chloroform vapour. They were counted and identified morphologically using taxonomic keys [11,12] and then desiccated over anhydrous copper sulphite and kept at room temperature until further processed. Abdomens of *An. gambiae *sensu lato were analysed by PCR for sibling species identification [13].

### Statistical methods

The simple expedient of adding one to each mosquito count in order to cater for zero counts can be misleading [14]. Therefore, Winbugs version^® ^1.4 was used to fit regression-based models to the data. The conceptual basis of this Bayesian regression has been described in detail elsewhere [15].

The following models were fitted to the data:

#### Scenario A: A linear model for sampling proportionality

*E*(*y*_*i*_) = *α*_*t*_*β*_*c*_*E*(*x*_*i*_)

Where: *E*(*y*_*i*_) is the expected number of mosquitoes caught using the method being tested; *E*(*x*_*i *_is the expected number of mosquitoes caught using the human landing method (assuming the same mosquito collector as bait); *α*_*t *_is a multiplication factor corresponding to trapping method *t *in relation to the reference trapping method which was human landing catch; and *β*_*c *_is a multiplication factor corresponding to human bait *c *compared to the reference catcher, assigned number 1, whose value is set to 1.

#### Scenario B: A non-linear model for sampling proportionality

*E*(*y*_*i*_) = *α*_*t*_*β*_*c*_(*E*(*X*_*i*_))^*yt*^

All the terms for model B are identical to model A except that it includes *y*_*t *_which is the exponent corresponding to trapping method *t*. A value of *y*_*t *_different from 1 indicates a lack of proportionality between the methods. Both models assumed Poisson errors in the numbers of mosquitoes caught by any of the three methods.

## Results

**Figure 1:**
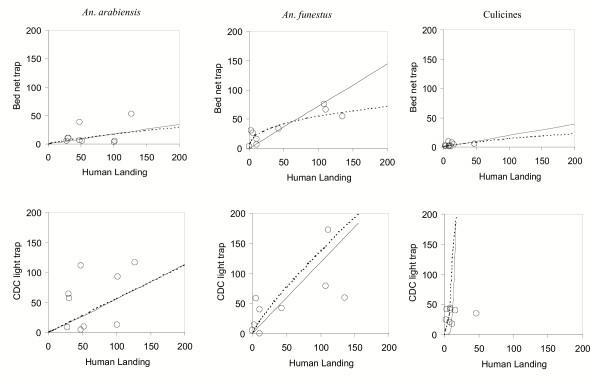
Numbers of female mosquitoes caught by the three sampling methods in 9 nights in western Kenya. Regression lines (unbroken) depict the fitted simple proportionality model (Model **A**) and the non-proportional (broken lines), density-dependent sampling efficiency model (Model **B**).

Overall, the Mbita trap, the human landing collection and the CDC light trap-bednet method caught 135, 576, and 474 *An. arabiensis *and 309, 427, and 470 *An. funestus *respectively. The corresponding figures for culicines mosquitoes (mainly *Culex *species) were 32, 121 and 578. Also, 30 male mosquitoes were caught, 29 of them by the CDC light trap and one in the landing collections. The parameter estimates from our models (Table 1, Figure 1) indicate the Mbita trap caught about 17%, 60%, and 20% of the number of *An. arabiensis*, *An. funestus*, and culicine species caught in the human landing collections respectively. There was consistency in the sampling proportionality between the Mbita trap and the human landing catch for both *An. arabiensis *and the culicine species whereas for *An. funestus*, the Mbita trap portrayed some density-dependent sampling efficiency. More specifically, the Mbita trap appears more sensitive than human landing catch at low mosquito densities. The CDC light trap, on the other hand, caught about 60%, 120%, and 552% of the number of *An. arabiensis*, *An. funestus*, and culicine species caught in the human landing collections respectively. There was consistency in the sampling proportionality between the CDC light trap and the human landing catch for both *An. arabiensis *and *An. funestus*, whereas for the culicines, there was no simple relationship between the CDC light trap catches and the landing catches (Table 1, Fig. 1). From PCR identification, all the successfully amplified specimens of *An. gambiae *s.l. were found to be *An. arabiensis*.

**Table 1:** Point estimates and 95% confidence intervals for model parameters.

		*An. arabiensis*	*An. funestus*	Culicines
Model A	*α*_*t*_: BNT versus HLC	0.17 (0.14, 0.21)	0.73 (0.62, 0.85)	0.20 (0.13, 0.29)
	*α*_*t*_: CDC versus HLC	0.56 (0.49, 0.66)	1.19 (1.03, 1.37)	4.84 (3.81, 6.21)
	*β*_*c*_: Person 2 vs person 1	0.38 (0.32, 0.45)	0.89 (0.77, 1.02)	0.54 (0.41, 0.72)
	*β*_*c*_: Person 3 vs person 1	0.54 (0.47, 0.62)	0.77 (0.66, 0.90)	0.59 (0.42, 0.80)
Model B	*α*_*t*_: BNT versus HLC	0.80 (0.52, 1.12)	0.39 (0.30, 0.50)	0.71 (0.00, 2.02)
	*α*_*t*_: CDC versus HLC	1.04 (0.79, 1.42)	0.84 (0.66, 1.06)	5.52 (3.08, 7.46)

## Discussion

The results obtained from this study indicate a three-fold decrease in efficiency for both the Mbita trap and the CDC light trap when used to sample *An. arabiensis *compared to their reported performance for nearby *An. gambiae *s.l. population that comprised of roughly equal numbers of *An gambiae sensu stricto and An. arabiensis *[6]. However, the consistency in the proportionality of their catches relative to the human landing collections was maintained. Several factors might explain this observation. First, this area is dominated by *An. arabiensis*, a mosquito species that is usually largely zoophagic but endophilic [16]. Therefore, protecting all the people in a bednet, as was the case in the houses where the Mbita trap and the CDC light trap were used, might have prompted the indoor resting *An. arabiensis *to seek alternative hosts outdoors. For the case of the human landing collection, the human bait was more readily available for such indoor resting mosquitoes.

Second, unlike in Lwanda [6], where no cattle were present in any of the homesteads that were sampled, large numbers of cattle were present in all the homesteads sampled at Ahero. Therefore, there was an alternative source of blood meal to this more flexible species, which can utilize both domestic [16] and wild bovids [17]. The availability of cattle could possibly account for the reported poor performance of the Mbita trap in sampling *An. arabiensis *in the highlands of Madagascar [7]. Other studies in Ahero have reported similar CDC light trap to human landing catch ratios [1] for *An. arabiensis *as this study found but with no correlation between the two methods.

The performance of the Mbita trap relative to the human catch for *An. funestus *in Ahero was similar to that reported for Lwanda [6,7] but showing density dependent sampling efficiency as density increased. Specifically, it appears that the Mbita trap may be more sensitive at low densities (Figure 1). It was considered whether this could be caused by the lowered attentiveness of individuals conducting tedious human landing catches when few mosquitoes are present. However, this was not found to be the case as the sampling efficiency of the CDC light trap, relative to the human landing catches showed no density dependence. The efficiency of the CDC light trap for *An. funestus *was about 2.5-fold that in Lwanda. The relatively high densities of this species in Ahero compared to Lwanda, might, at least partly, account for these observations. At Lwanda, where the densities of *An. funestus *were low, no density-dependent sampling efficiency was noted for the Mbita trap while some density-dependent sampling efficiency was noted for the CDC light trap [6] suggesting that the Mbita trap is more sensitive in low densities while the CDC light trap is better at higher densities of this species.

Many studies have evaluated the performance of the CDC light trap relative to the human landing catch but it is very difficult to compare the results due to the different methodologies and sampling procedures applied. In this study, the three methods were used concurrently in different houses on the same night while in other studies [2,18,19] the methods were used in the same houses but on different nights. Small differences in sleeping arrangements, availability of alternative hosts, temperatures, humidity, and wind speed and direction between the different days might introduce some sampling bias in this case. Furthermore, the procedures used for conducting human landing catch also vary appreciably: some studies have used one human per house to perform landing catches [20] while others [2,18] have used two catchers in the same house. There is, therefore, a need to standardize the operational conditions and sampling procedures used if valid comparisons between various studies in are to be made

## Conclusions

Although the Mbita trap is less sensitive than either the human landing collections or the CDC light trap, for a given investment of time and money [5], it is likely to catch more mosquitoes over a longer period, larger number of sampling sites or both. Adult mosquito densities are highly aggregated in space and time, resulting over 80% of transmission occurring in 20% of places and time [21], and the importance of catching them across large numbers of sampling points and frequent intervals to obtain representative samples of the vector population has recently been emphasized [22]. The Mbita trap may therefore be very useful for enabling community members in collecting large numbers of samples that are representative of the overall vector population at a less cost [5] than a smaller number of light traps/human catchers. Used in this way, rather than as a direct replacement for the CDC light trap-bednet method, this trap will surely find a place in community-based malaria vector surveillance. It might be important to note that some community members in Rusinga, an island adjacent to ICIPE-Mbita point where the trap was developed, have adopted this trap for passive mosquito surveillance with some encouraging results. However, this trap might not work in all epidemiological settings [7] and therefore more mosquito behavioural studies should be carried out in order to gain more insight to guide further development of mosquito sampling and control tools. The human landing catch should be maintained as the standard reference method for use in calibrating new methods for sampling the human biting population of mosquitoes.

## Authors' contributions

EMM designed the trap, designed the study and drafted the manuscript. GOM & DOO supervised the fieldwork and recorded the data. LWI & PNN were involved in the study design and drafting the manuscript, TAS carried out the data analysis and helped in the interpretation of results. GFK guided the experimental design, data analysis and drafting of manuscript. BGJK conceived the initial idea of developing the trap and solicited for funds used in the trap development and trials. All authors read and approved the final manuscript.
